# Development and In-House Validation of an Enzyme-Linked Immunosorbent Assay and a Lateral Flow Immunoassay for the Dosage of Tenofovir in Human Saliva

**DOI:** 10.3390/bios13060667

**Published:** 2023-06-20

**Authors:** Simone Cavalera, Thea Serra, Antonio Abad-Fuentes, Josep V. Mercader, Antonio Abad-Somovilla, Fabio Di Nardo, Antonio D’Avolio, Amedeo De Nicolò, Valentina Testa, Matteo Chiarello, Claudio Baggiani, Laura Anfossi

**Affiliations:** 1 Department of Chemistry, University of Turin, 10125 Turin, Italy; simone.cavalera@unito.it (S.C.); thea.serra@unito.it (T.S.); fabio.dinardo@unito.it (F.D.N.); v.testa@unito.it (V.T.); matteo.chiarello@unito.it (M.C.); claudio.baggiani@unito.it (C.B.); 2 Institute of Agricultural Chemistry and Food Technology, Spanish Council for Scientific Research (IATA-CSIC), 46980 Paterna, Valencia, Spain; aabad@iata.csic.es (A.A.-F.); jvmercader@iata.csic.es (J.V.M.); 3 Department of Organic Chemistry, University of Valencia, 46100 Burjassot, Valencia, Spain; antonio.abad@uv.es; 4 Unit of Infectious Diseases, Department of Medical Sciences, University of Turin, 10126 Turin, Italy; antonio.davolio@unito.it (A.D.); amedeo.denicolo@unito.it (A.D.N.)

**Keywords:** point-of-care testing, therapeutic drug monitoring, anti-retroviral drugs, adherence, human immunodeficiency virus

## Abstract

Highly active antiretroviral therapy (HAART) includes very potent drugs that are often characterized by high toxicity. Tenofovir (TFV) is a widely used drug prescribed mainly for pre-exposure prophylaxis (PreP) and the treatment of human immunodeficiency virus (HIV). The therapeutic range of TFV is narrow, and adverse effects occur with both underdose and overdose. The main factor contributing to therapeutic failure is the improper management of TFV, which may be caused by low compliance or patient variability. An important tool to prevent inappropriate administration is therapeutic drug monitoring (TDM) of compliance-relevant concentrations (ARCs) of TFV. TDM is performed routinely using time-consuming and expensive chromatographic methods coupled with mass spectrometry. Immunoassays, such as enzyme-linked immunosorbent assays (ELISAs) and lateral flow immunoassays (LFIAs), are based on antibody–antigen specific recognition and represent key tools for real-time quantitative and qualitative screening for point-of-care testing (POCT). Since saliva is a non-invasive and non-infectious biological sample, it is well-suited for TDM. However, saliva is expected to have a very low ARC for TFV, so tests with high sensitivity are required. Here, we have developed and validated a highly sensitive ELISA (IC50 1.2 ng/mL, dynamic range 0.4–10 ng/mL) that allows the quantification of TFV in saliva at ARCs and an extremely sensitive LFIA (visual LOD 0.5 ng/mL) that is able to distinguish between optimal and suboptimal ARCs of TFV in untreated saliva.

## 1. Introduction

Tenofovir (TFV) is a successful and long-lasting antiretroviral (ARV) drug commonly used to treat human immunodeficiency virus (HIV) and hepatitis B virus (HBV) and for preexposure prophylaxis, either alone or in combination with other drugs. TFV is included in many fixed-dose formulations of highly active antiretroviral therapies (HAART), which have dramatically reversed the trend in HIV infection and AIDS-related deaths since their introduction in 1996. Nowadays, TFV is more frequently administered as the disoproxil fumarate (TDF) [1] salt, although recently, the alafenamide (TAF) [2,3] salt has also been introduced. A strict therapeutic administration of TFV (300 mg/day for TDF, 30 mg/day for TAF) must be observed to avoid toxic or sub-optimal levels in the organism [4,5,6,7,8,9]. Drug overdose leads to high levels of plasmatic TFV, which is inherently harmful to the kidneys and bones. Renal dysfunction results in an increase in serum creatinine levels, a decrease in glomerular filtration rate, proteinuria, glycosuria, a decrease in plasmatic phosphate, and an increase in alkaline phosphatase. The TAF formulation may help in this regard because its therapeutic dose is 10-fold lower compared to TDF due to its higher cell-penetration capacity, and the residual amount of free TFV in plasma is lower. On the other hand, suboptimal TFV dosing increases the risk of disease relapse as well as the emergence of drug resistance, leading to treatment failure. Therefore, the adherence requirement for a successful management of the infection has been estimated to be 95%, and this is the actual bottleneck of the effectiveness of the HAART. The mismanagement of the ARV drug regimens leads rapidly to therapeutic failure through poisoning and the development of lethal and infectious drug-resistant viral strains [10]. Noncompliance is widely recognized as a major cause of HAART failure [11]. Since the non-compliant behavior is associated with unpredictable causes and factors (misunderstanding of complicated regimens, refusal of therapy because of psychological and physical side effects, carelessness, etc.), it is almost impossible to prevent by a priori interventions [11,12,13,14]. In addition, even if the administration is carried out correctly, the inter-patient variability in metabolism can still affect how well a dose works. This becomes even more important when the therapeutic administration range is limited. To reduce treatment failures, it is strongly advised to use therapeutic drug monitoring (TDM) in biological fluids to evaluate patient adherence and track the effectiveness of the dose that was delivered. HPLC-based methods, often coupled with MS, are commonly used for TDM of TFV. These methods are time-consuming, costly, and poorly suited for real-time testing outside laboratories, as in most developing countries. TDM of TFV generally involves HPLC-based methods, often coupled with MS detection, that are time-consuming, costly, and poorly suitable for real-time testing in non-laboratory settings, such as in most of the developing countries. Immunoassays are based on the highly specific molecular recognition between antigens and antibodies. For detecting small molecules such as TFV, a competitive immunoassay format is applied, where an antigen competes with the analyte for the binding to a specific antibody. As a result, the signal intensity is inversely proportional to the concentration of the analyte because the analyte prevents the antibody from binding to the competing antigen. Enzymatic immunoassays, such as ELISA (Enzyme-Linked ImmunoSorbent Assay), typically perform sensitive and selective quantification in a few hours without the need for expensive equipment, and mostly on untreated biological samples. The lateral flow immunoassay (LFIA) technique has been widely recognized as one of the most successful and widespread diagnostic methods for the on-site screening of infectious diseases. LFIA devices are also employed for the detection of small molecules such as hormones, toxins, drugs of abuse, residual antibiotics, and other biomarkers [15]. Since LFIA meets all the ASSURED (Affordable, Sensitive, Specific, User-friendly, Rapid/Robust, Equipment-free, Deliverable) criteria recommended by the World Health Organization (WHO) in 2012 [16], it has been suggested as the most strategic tool for POC testing. A typical LFIA device is a portable cassette including a strip composed of overlayered materials containing all the reagents required for the test. The device allows the direct detection of the target molecule by exploiting the affinity and specificity of the antibody–antigen interaction [17,18]. A LFIA can be completed in 5–15 min, and the results are typically colorimetric and simple to read with the naked eye, which speeds up decision making and intervention [19]. As for ELISA, LFIA is inherently competitive when dealing with small molecules, so color intensity is inversely proportional to the analyte concentration. Usually, LFIA is intended for qualitative analysis. In the context of TDM, this means that a cut-off value for the drug concentration has to be defined to discriminate compliant from non-compliant subjects (i.e., subjects with the desired drug concentration and subjects showing lower levels). The visual limit of detection (vLOD) of the device must be tailored so that the color of the test line vanishes at that concentration of analyte. TDM of TFV is performed on blood samples using conventional methods, which requires invasive sample collection and, in the case of HIV-positive individuals, poses a risk of infection to the physician. Unfortunately, except for TFV levels in urine after TDF administration established in the TARGET study made by P.K. Drain et al. in 2019, there is still no reliable correlation between adherence to therapy and concentration of TFV in other biological fluids. Since TFV is relatively concentrated in urine, a thorough investigation into the dose-adherence correlation could be conducted. The reported urinary level to discriminate therapy adherence is 6.48 µg/mL (interquartile range: 3.94–14.3) [20]. Although minimally invasive, urine collection does not preclude counterfeit, because it requires the subject’s cooperation, and a physician cannot be present during sampling. LFIA can be performed on almost any kind of liquid sample, including milk, blood, serum, saliva, urine, beverages, and water. Saliva is potentially the most appropriate kind of sample for the follow up of HIV-positive subjects because it is less infective than blood and can be collected by either the patient or the physician. De Lastours et al. determined in 2011 that the salivary levels of TFV in TDF-treated (perfectly adherent) patients ranged between 0.4 and 25 ng/mL, with a mean value of 2.75 ng/mL. The lower TFV level in saliva compared with urine is because TFV has a very low saliva-to-plasma transfer ratio (3 ± 4%), as reported by de Lastours et al. and later confirmed by the IPERGAY study by Fonsart et al. in 2017 (2%) for pre-exposure prophylaxis (PrEP) with a single dose of tenofovir disoproxil fumarate [21]. After administration of TAF at the recommended dose, TFV levels are expected to be approximately 10-fold lower, thus requiring extreme sensitivity for detection of TFV in patients treated with TAF. In a previous study, we synthesized bioconjugates and generated monoclonal antibodies to develop a lateral flow prototype capable of dosing TFV in urine samples [22]. The test demonstrated extreme sensitivity (vLOD 1.4 ng/mL), largely exceeding the requirements for the application.

**Figure 1:**
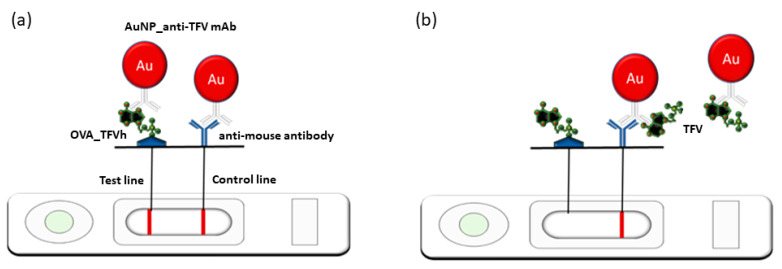
Scheme of the interpretation of the LFIA for TFV detection: (**a**) two lines are visible for samples from non-adherent subjects (the concentration of salivary TFV below the cut-off value and, therefore, the anti-TFV mAb labeled with AuNP stacks to the competing antigen; (**b**) just one line (the control line) is visible for a sample containing high levels of TFV because binding of the AuNP-mAb complex to the antigen in the test line is inhibited by the analyte.

In this work, using those in-house-produced immunoreagents, we developed and validated a competitive enzyme-linked immunosorbent assay (cELISA) for the dosage of TFV in saliva samples. The salivary assessment of therapeutic compliance would disclose the quantitative analysis even in non-laboratory settings, and reduce the opportunity to counterfeit the analysis, since saliva collection can be carried out under supervision, contrarily to urine sampling. On the other hand, the sensitivity required for TFV assessment in saliva is very demanding. The checkerboard strategy was used to optimize the concentrations of the antigen on the microplate, the anti-TFV antibody, and the secondary enzyme-labeled antibody, as well as the composition of working and washing buffers and the proper dilution factor of the salivary matrix. In the absence of a consensus cut-off value, we identified 0.3–2.8 ng/mL as the range of salivary TFV most likely indicative of therapy adherence for patients receiving TDF and TAF. Then, we evaluated the potential of the newly developed cELISA by determining its half-maximal inhibitory concentration (IC50), dynamic range, coefficient of variation (CV%), and cross-reactivity (CR%) with other drugs and similar molecules. Furthermore, we developed and validated an LFIA for the detection of TFV in saliva. In addition to the advantages reported for the ELISA testing with respect to HPLC-MS/MS, the LFIA adds the advantage of a low-cost and user-friendly qualitative screening for a large number of samples, in real-time, according to the WHO recommendations. The anti-TFV mAb was directly adsorbed onto the surface of gold nanoparticles (AuNPs), while a conjugate of TFV with ovalbumin (OVA-TFVh) was immobilized onto the nitrocellulose membrane (NC) and used as the competing antigen (Figure 1). An anti-mouse immunoglobulin polyclonal antibody from rabbit was used as the control line, which captured the labeled mAb regardless of the presence of the analyte. The optimal amount of mAb adsorbed onto the AuNPs, the concentration of the competing antigen on the test line, and the behavior of the materials and their pre-treatment to limit the matrix effect were studied. The IC50 and visual limit of detection, which corresponded to the cut-off for qualitatively assessing optimal and sub-optimal concentrations of TFV in the saliva samples, were used to evaluate the analytical performance of the LFIA.

## 2. Materials and Methods

### 2.1. Competitive ELISA (cELISA) for TFV in Buffer

This cELISA format was carried out with the immobilized conjugate and indirect antibody detection [23]. Microplate wells were coated with 150 µL per well of OVA-TFVh [22] solution in 50 mM carbonate buffer, pH 9.6, by overnight incubation at 4 °C. Plates were washed three times with washing solution (milliQ H_2_O with 0.05% (*v*/*v*) Tween 20) after each incubation step. To block the residual surface of the wells, 300 µL per well of 0.5% (*w*/*v*) casein in 20 mM phosphate buffer, pH 7.4, containing 0.05% (*v*/*v*) Tween20 was used as an overcoating buffer, and the plates were incubated for 1 h at RT and then washed. The calibration curve for competitive immunochemical reaction was carried out by adding 200 µL per well of TFV (28.72–11.50–2.87–1.43–0.57–0.29–0.14–0 ng/mL) and the anti-TFV mAb solution in 20 mM phosphate buffer, pH 7.4, containing 0.05% (*v*/*v*) Tween20. The mixture was incubated for 1 h at 37 °C and the plates were washed. The fraction of bound mAb was detected by adding 200 µL per well of peroxidase-labeled rabbit anti-mouse immunoglobulin polyclonal antibody (RAM-HRP) in 20 mM phosphate buffer, pH 7.4, containing 0.05% (*v*/*v*) Tween20, 0.1% (*w*/*v*) casein, and 0.13 M NaCl. The plates were incubated at RT for 1 h, and after washing as before, 200 µL per well of TMB chromogenic substrate solution was added. The color due to TMB oxidation was stopped after 30 min of incubation by adding sulfuric acid (2 M) and was measured at 450 nm. A full-factorial experimental design was made to define the concentration of competing antigen OVA-TFVh, anti-TFV mAb, and RAM-HRP providing a sufficient maximum signal (Amax) and IC50. OVA-TFVh and anti-TFV mAb were used at 400, 100 and 40 ng/mL, and the RAM-HRP was diluted 5000-fold and 10,000-fold. The calculations were made by means of the Chemometric Agile Tool, free software [24]. TFV-spiked saliva samples were measured by diluting the matrix with the anti-TFV mAb solution. Absorbance was plotted against the TFV concentration, and the parameters of the four-parameter logistic equation [25] were extracted by means of SigmaPlot 14.0 software (Systat, Palo Alto, CA, USA).

### 2.2. Development and Analytical Validation of the cELISA in Saliva

The ELISA protocol was implemented until an effective method for detecting TFV in saliva was found. The matrix effect minimization was addressed by changing the sample dilution (1/2, 1/4, 1/8, 1/16, 1/25), buffer pH (6.4, 7.4), and additives (casein: zero, 0.5, 1%; Nalco: 0.15, 0.3, 0.6, 1 M), and the number of washings (3, 5). The in-house validation of the method included the estimation of precision, accuracy, recovery, and dynamic range. The imprecision was estimated by measuring the inter- and intra-assay reproducibility and was calculated by analyzing each calibrator level in four replicates on the same day and on three days (12 replicates). The concentration of TFV estimated from each replicate was calculated by following the back-calculation approach detailed in Di Nardo et al. [26]. The overall imprecision at each level was calculated as the mean CV% values. The limit of detection (LOD) and the upper and lower limits of quantification (LLOQ and ULOQ) were defined based on the error curve, as the TFV levels that can be measured with an acceptable inaccuracy of 20% and 15%, respectively [27]. The recovery test was conducted by using a pool of saliva samples collected from volunteers spiked at three levels corresponding to the threshold assuring perfect compliance, the 2× threshold, and the 0.5× threshold. Recovery was estimated as the quotient between the TFV concentration and the fortification concentration ×100.

### 2.3. Monoclonal Antibody Labeling with Gold Nanoparticles

AuNPs with a mean diameter of 32 nm (LSPR maximum wavelength 525.5 nm) were synthesized following the citrate reduction method as previously reported [28]. The conjugation of anti-TFV mAb to AuNPs was conducted by passive adsorption in basic medium, as previously described. Briefly, the pH was adjusted to 8 with 50 mM carbonate buffer, pH 9.6. Then, for each milliliter of AuNP suspension (ca., optical density 1), different amounts of the mAb were added starting from the value defined according to the salt-induced aggregation test [29]. The salt-induced aggregation test was carried out as follows: 250 μL of AuNP solution at optical density (O.D.) 1 was inserted into wells of a microtiter plate and incubated for 30 min with increasing amounts (0–2.5 μg) of the anti-TFV mAb. Then, 25 μL of aqueous NaCl (10% *w*/*v*) was added and reacted for 10 min to promote the aggregation of unstable AuNPs. The absorbance of the solutions was read at 540 nm and 620 nm by a Multiskan FC, Microplate Photometer (Thermo Scientific, Waltham, MA, USA) and the ratio between the unaggregated and aggregated fraction was plotted against the amount of the mAb [29]. The solution was gently stirred and left to react for 30 min at 37 °C. Then, 100 µL of 1% (*w*/*v*) BSA in 38 mM borate buffer, pH 8, was added and reacted under gentle stirring for 10 min. The AuNP-mAb conjugate was recovered by centrifugation (10 min at 7100× *g*), washed twice with 0.1% (*w*/*v*) BSA in 38 mM borate buffer, pH 8, and reconstituted in the storage buffer (38 mM borate buffer, pH 8, supplemented with 1% *w*/*v* BSA, 2% *w*/*v* sucrose, 0.05% *v*/*v* Tween20, and 0.02% *w*/*v* of sodium azide). The antibodies labeled with AuNPs were stored at 4 °C until use.

### 2.4. The LFIA Strip Preparation

Strips were prepared from nitrocellulose (NC) membranes (MHF180 plus card) employing an XYZ3050 platform (Biodot, Irvine, CA, USA). In detail, a solution of antigen OVA-TFVh (0.5 mg/mL) diluted in phosphate buffer (20 mM, pH 7.4) was dispensed to form the test line, while the anti-mouse antibody from rabbit (RAM) (0.3 mg/mL), diluted in phosphate buffer, formed the Control line. Reagents were dispensed at a flow rate of 1 μL/cm, keeping a distance of 5 mm between the lines. The conjugate pad was previously saturated with the conjugate storage buffer and dipped into a labeled anti-TFV antibody solution at optimal optical density (OD = 2) and dried for 3 h at room temperature, protecting from light and dust. The sample pad (GFBR4) was previously saturated with the conjugate storage buffer. NC membranes were dried at 37 °C for 60 min under vacuum, layered with the sample, conjugate, and adsorbent pads (Figure 1), cut into strips (5 mm width) using a CM4000 guillotine (Biodot), and inserted into plastic cassettes (Kinbio, Shangai, China) to obtain stand-alone LFIA devices. Cassettes were stored in the dark in plastic bags containing silica at room temperature until use.

### 2.5. The LFIA Test Procedure

The LFIA for TFV was carried out at room temperature. An amount of 90 μL of blank and fortified saliva sample was dispensed on the sample pad through the sample well to start the capillary flowing of the solution toward the adsorbent pad. After 10 min, the results were visually estimated, as shown in Figure 1. The signals generated at the test and control lines, due to AuNP-mAb binding to immobilized bioreagents, were measured by acquiring the images of the LFD by a portable scanner (OpticSlim 550 scanner, Plustek Technology GmbH, Norderstedt, Germany) and quantifying the intensity of the color on each line with QuantiScan 3.0 software (Biosoft, Cambridge, UK). The performance was compared according to the following points: (1) intensity of color of the test and control lines; (2) sensitivity of the LFIA for measuring TFV; and (3) cross-reactivity toward potentially interfering molecules. Accordingly, TFV calibrators at 0, 0.05, 0.25, 0.5, 1, and 1.5 ng/mL were prepared by fortifying a pool of human saliva. The signals produced at the test (T) and control (C) lines were singularly quantified, converted in T/C ratios, normalized for the signal of the blank saliva, and then plotted versus TFV concentration. The four-parameter logistic equation was used to estimate the IC_50_.

### 2.6. Saliva Samples

Saliva samples were collected at 1 pm by using the Salivette Swab (Salimetrics, LLC, Carlsbad, CA, USA) and following the supplier’s instructions. In detail, each subject was requested to rinse their mouth with water, wait for 10 min, and then put the swab under their tongue for 3 min. The swab was placed in the upper part of the collector and immediately frozen at −20 °C for at least 24 h. After thawing, saliva was recovered by centrifugation of the swab (15 min at 2000× *g*) and subsequently analyzed. More samples were collected on different days (at 1 p.m.) as described above and pooled for allowing the execution of several assays while minimizing matrix variability. Donors had never been in contact with any of the TFV-derived drugs or other anti-retrovirals. They were contacted and provided informed consensus about the use of their specimens. Fortification with TFV and analogs [30,31,32] for specificity assessment was performed by direct addition to the blank saliva pool.

## 3. Results and Discussion

### 3.1. Development of the cELISA for TFV in Buffer

The experimental design was carried to define the combination maximizing the inhibition capacity (low IC50) and a sufficient starting signal (Amax > 1). In Figure S1, the response surface of the ratio between the Amax and the IC50 values of the two checkerboard titrations obtained by using two dilutions of RAM-HRP is reported. The grey zone indicates the surface area where Amax is lower than the acceptable value. At the lower dilution of RAM-HRP (1:5000), half of the response surface showed an acceptable Amax, and the higher Amax/IC50 ratio was in the upper left corner of the experimental space (purple circle). Considering the higher dilution of RAM-HRP (1:10,000), none of the experiments provided sufficient Amax, so these data are not shown. Consequently, a 40 ng/mL solution of the immobilized competing conjugate OVA-TFVh was used in combination with a 400 ng/mL solution of anti-TFV mAb. Under these conditions, the calibration curve in the buffer resulted in Amax = 1.6 a.u. and IC50 = 0.14 ng/mL.

### 3.2. Application of the cELISA to Saliva

**Figure 2:**
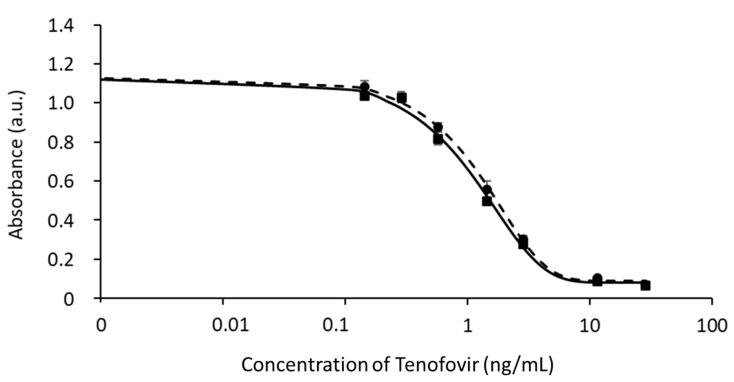
Comparison of curves obtained by diluting TFV in the predilution buffer (squares, full line) and in saliva (1:25 in working buffer, rounds, dotted line).

Saliva is an extremely variable matrix that typically has a significant impact on ELISA performance. The proneness of cELISA to matrix interference was investigated by carrying out calibration curves in which TFV calibrators included a variable quantity of saliva. Inhibition curves were plotted toward TFV concentration and the Amax and IC50 values were compared to those obtained for TFV calibrators prepared in the buffer (Figure S2). As the presence of small amounts of saliva showed a large impact on the signals and assay sensitivity, the composition of the buffer used to prepare calibrators was studied in order to mimic the sample interference. To this aim, the pH, ionic strength, and additives were investigated. In parallel, the dilution factor and the composition of the diluent of saliva were also studied. A pool of blank saliva samples was fortified with TFV at different TFV levels. The condition chosen as optimal was considered the one providing the maximal accuracy for measuring TFV in saliva by a standard curve prepared in buffer and resulted to be standards of TFV dissolved in a predilution buffer (20 mM phosphate buffer, pH 7, 630 mM NaCl, 0.05% Tween20, 1% *w*/*v* casein) and 1:25 dilution of salivary samples with the working buffer (20 mM phosphate buffer, pH 7.4, containing 0.05% (*v*/*v*) Tween20, 0.1% (*w*/*v*) casein, 0.13 M NaCl). Under these conditions, the samples fortified with TFV overlapped with the standard curve (Figure 2). The new calibration curve showed A_max_ = 1.1 a.u. and IC_50_ = 1.2 ng/mL. IC_10_–IC_90_ comprised between 0.4 and 8.6 ng/mL.

### 3.3. Analytical Validation of the cELISA Method for TFV in Saliva

The cELISA was validated by assessing the recovery, inter- and intra-assay variability, and quantification range (lower limit of quantification, LLOQ; upper limit of quantification, ULOQ). Three levels of fortification were used for the recovery test, i.e., one below, one near, and one above the cut-off reference level for assessing the adherence to therapy with TFD (TFV 1.3, 2.7, and 5.4 ng/mL, respectively). The CV% values were below 15% and recovery values ranged from 73.4 ± 0.6% to 133.6 ± 11.1% (Table 1). Precision was measured for all levels used in the calibration curve by repeating the curve in quadruplicate on three days to evaluate the inter- and intra-assay variability (Table 2). By plotting the error curve (Figure S3), LOD was considered the TFV concentration that can be measured with an acceptable error (less than 20%) [26,27,33,34,35,36,37,38,39]. The LOD resulted as 0.25 ng/mL. The accuracy, measured over the entire series of measurements, ranged from 85.8% to 145.6%. The within-method variability caused by the saliva appeared to be better randomized by testing on different days than by testing replicates in a single assay. The quantification range was defined as 0.4–16 ng/mL (<15% of imprecision from back calculation).

### 3.4. Development of an LFIA for Detecting TFV in Saliva

**Figure 3:**
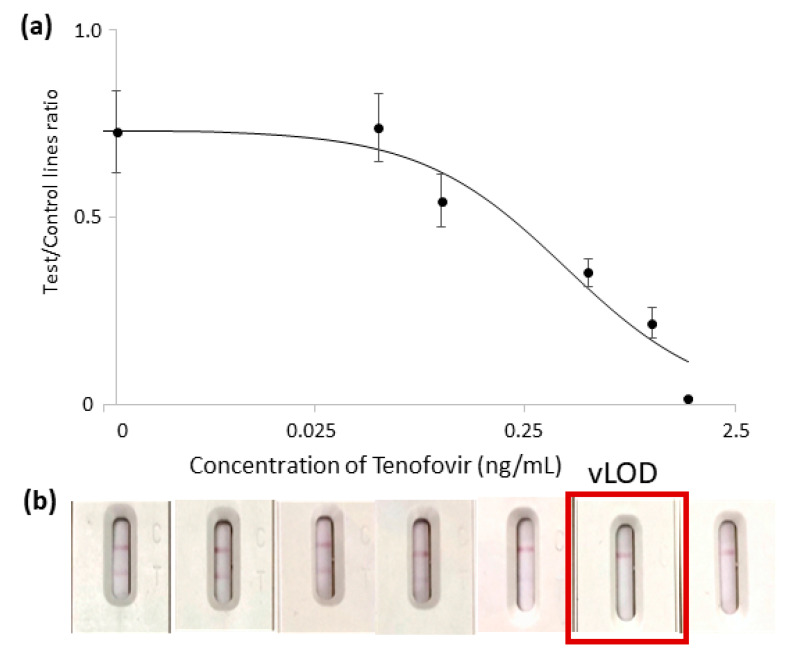
Standard TFV-fortified saliva curve (0, 0.05, 0.25, 0.1, 0.5, and 1.5 ng/mL) (**a**). The color intensity at the test line (T) was normalized with respect to the intensity at the control line (C) and plotted against TFV concentration. Pictures of devices corresponding to the calibrators are shown in (**b**) and the visual limit of detection (test line disappearance) is highlighted.

The LFIA technique has been applied to many different biological fluids; however, the complexity of the matrix is a critical point in the development of LFIA devices. To study the application of LFIA to saliva, some strategies (addition of casein to the buffers, large dilution factors of the sample, pre-saturation of materials composing the strip) have been used to avoid destabilization of the probe caused by the mucins present in the oral fluid, typically resulting in a lower color and partial aggregation of the nanoparticles during the assay. Notwithstanding the fact that the addition of casein to buffers excessively attenuated the coloring of the test line and that dilution is not preferable, due to loss of sensitivity, the pre-saturation of the sample pad with the conjugate storage buffer was sufficient to properly re-suspend the probe and avoid destabilization. This aspect was particularly convenient because it allowed the use of undiluted saliva as a sample without further compromising the sensitivity. Regarding the stability of the gold nanoparticles, the salt-induced aggregation test revealed that 8 µg of anti-TFV mAb was required to stabilize the nanoparticles from saline stress (Figure S3). Based on this value and taking into account that the lower the antibody amount, the better the sensitivity of competitive immunoassay [40,41], 2, 4, and 8 µg were used to coat the AuNPs. An amount of 2 µg of anti-TFV mAb was found to provide a better IC50 value while giving sufficient color in the absence of TFV. An uncoated AuNP surface was passivated by BSA to avoid aggregation. The matrix-matched TFV calibrators were prepared as described in the previous section for the analytical validation of ELISA. The four-parameter logistic equation was chosen as the mathematical fitting model for experimental points. Despite observing a limited reproducibility for samples containing extremely low levels of TFV, the curve showed that the signal varied coherently with the expected behavior (inverse correlation to TFV). The LFIA was not intended for quantitative purposes but for classifying samples as compliant (undetectable color at the test line) or uncompliant (clearly visible signal at the test line). The visual LOD (vLOD), defined as the TFV level that gave the complete disappearance of the signal, was established at 0.5 ng/mL, as can be seen in Figure 3. This value corresponded to the lower limit of the reference level measured by de Lastours et al. [42] (0.4–25 ng/mL, average 2.75 ng/mL) for perfect adherence to TDF-administered patients. Therefore, the prototype was able to identify all subjects with TFV salivary levels indicating scarce adherence to the therapy. On the other hand, TFV salivary levels related to TAF administration are expected around 10-fold lower (tens-thousands of pg/mL) and would require even higher sensitivity.

The stability of the LFIA over time was assessed via accelerated stability studies to simulate the degradation of the devices [43,44]. The performance of the LFIA was evaluated after three and seven days of storage at 37 °C by analyzing the pool of blank saliva unfortified and fortified at 0.3, 0.6, and 1.4 ng/mL of TFV to compare the color of the test line for the blank sample and the vLOD of the method. The performance did not vary significantly, according to the one-way ANOVA (*p*-value > 0.05, Table S1).

### 3.5. Selectivity of the LFIA in Saliva

**Figure 4:**
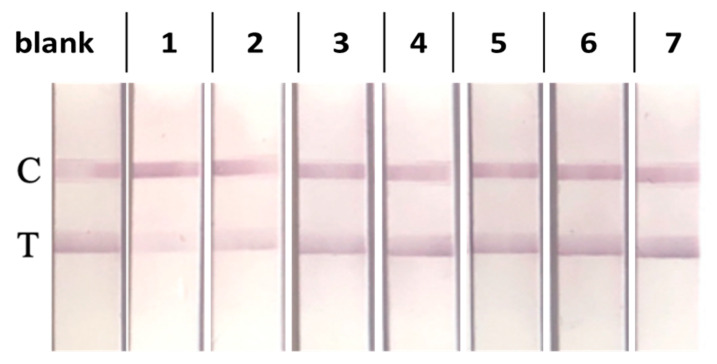
(From left to right) LFIA visual results of analyzing blank saliva; saliva fortified with 1000 nM of TDF (1), TAF (2), DTG (3), and EVG (4); and 100 nM of ATP (5), adenine (6), and caffeine (7).

The selectivity of the standalone LFIA test toward the two TFV-based pro-drugs (TDF and TAF), two other antiretrovirals (Dolutegravir, DTG and Elvitegravir, EVG), and three molecular analogs that can be commonly found in saliva (adenosine triphosphate, ATP; adenine; and caffeine) was checked in saliva by means of single-point measurement by both visual evaluation and signal quantification. The visual results are shown in Figure 4. Among the investigated substances, only TDF and TAF showed measurable CR%, though lower than 1% (Table 3).

## 4. Conclusions

The high affinity shown by the anti-TFV mAbs [41], as also reported in a previous work, largely exceeded those of the previous rapid analytical tests and allows for the detection of TFV in an important matrix, such as saliva, where the levels of TFV are very low. Thanks to this, we in-house-validated a cELISA method and developed a rapid LFIA for the detection of TFV in saliva. The developed ELISA method is the first represented for TFV drug monitoring in saliva and is the most sensitive for the detection of TFV, to the best of our knowledge. TFV is usually quantified by liquid chromatography coupled to tandem mass spectrometry. As an example, Fonsart et al. reported the development and validation of an HPLC-MS/MS method [21] that ameliorated the one previously described in De Lastours et al. [40]. The HPLC-MS/MS method showed an LLOQ of 1 ng/mL for plasma and 0.25 ng/mL for saliva. Similarly, the few works devoted to measuring TFV in saliva showed limits of detection at the ng/mL level [45]. Therefore, the newly developed ELISA demonstrated a sensitivity very close to the one shown by much more sophisticated approaches. As a further application, we developed the first LFIA for the on-field detection of TFV in the salivary matrix. The selectivity in terms of CR% was measured (0.8% for TDF as the highest) for seven potentially interfering molecules. These performances appear as suitable for TDF adherence assessment (IC50 = 1.2 ng/mL and vLOD = 0.5 ng/mL), while for TAF administration, the expected adherence-related salivary level could not be reached by the sensitivity of the developed devices. Notwithstanding, TAF can be evaluated in other biological fluids, where the concentrations match the sensitivity of the developed assays. Further studies aimed at evaluating the clinical performance of the two immunoassays developed here will be needed. However, provided their figures of merit, the two methods become to be used for screening (LFIA)–confirmatory (ELISA) monitoring of TFV, especially in low-resource settings, where a less expensive, complicated, and time-consuming approach compared to HPLC-MS/MS-based methods is required. Furthermore, the intrinsic multiplexing capability of the LFIA platform envisages the combined detection of TFV with other biomarkers to enhance the diagnostic and prognostic value of the assay.

## Figures and Tables

**Table 1:** Recovery and coefficients of variation for three fortification levels of TFV in the salivary matrix.

TFV Added (ng/mL)	TFV Mean Concentration ± SD ^a^ (ng/mL)	Mean Recovery ± SD ^a^ (%)	CV (%)
1.3	1.0 ± 0.1	73.4 ± 0.6	8.2
2.7	2.9 ± 0.3	106.8 ± 11.9	11.1
5.4	7.2 ± 0.6	133.6 ± 11.1	8.3

^a^ *n* = 3.

**Table 2:** Repeatability and reproducibility of TFV quantification in salivary matrix.

TFV (ng/mL) ^a^	Intra-Assay (%), *n* = 4	Inter-Assay (%) *n* = 3	Accuracy (Mean *n* = 4 × 3)
28.7	9.5	9.5	85.8
11.5	9.6	4.2	120.3
2.9	13.4	3.5	99.0
1.4	24.0	3.1	96.7
0.6	49.6	11.6	106.4
0.3	63.4	28.2	111.2
0.1	75.5	17.0	145.6

^a^ 28.72 ng/mL = 100 nM.

**Table 3:** The CR% calculated for the TFV analogs and co-administered drugs. The values were calculated as the percentage ratio between the actual concentration of the compound and the one extracted from the calibration 4-parameter logistic equation.

Compound	CR%
TDF	0.8%
TAF	0.1%
DTG	<0.003%
EVG	<0.003%
ATP	<0.03%
Adenine	<0.03%
Caffeine	<0.03%

## Data Availability

Not applicable.

## Supplementary Materials

The following supporting information can be downloaded at: https://www.mdpi.com/article/10.3390/bios13060667/s1, Materials and Chemicals, AuNP synthesis, Salt-induced aggregation test; Figure S1: Response surface of DoE for ELISA development, Figure S2: Matrix effect graph; Figure S3: Salt-induced aggregation test results.
