# Supplementary material for: Development and In-House Validation of an Enzyme-Linked Immunosorbent Assay and a Lateral Flow Immunoassay for the Dosage of Tenofovir in Human Saliva
Source: Biosensors (Basel). 2023 Jun 20;13(6):667. doi: 10.3390/bios13060667 (PMC10296389; doi:10.3390/bios13060667)
Supplement: Supplementary file 1 [file biosensors-13-00667-s001.zip › biosensors-2413593-supplementary.pdf]

biosensors

MDPI

Supporting Information File

Development and In-House Validation of an Enzyme-Linked Immunosorbent Assay and a Lateral Flow Immunoassay for the Dosage of Tenofovir in Human Saliva

Simone Cavalera [1], Thea Serra [1], Antonio Abad-Fuentes [2], Josep V. Mercader [2], Antonio Abad-Somovilla [3], Fabio Di Nardo [1], Antonio D'Avolio [4], Amedeo De Nicolò [4], Valentina Testa [1], Matteo Chiarello [1], Claudio Baggiani [1] and Laura Anfossi [1,]*

[1] Department of Chemistry, University of Turin, 10125 Turin, Italy; simone.cavalera@unito.it (S.C.); thea.serra@unito.it (T.S.); fabio.dinardo@unito.it (F.D.N.); v.testa@unito.it (V.T.); matteo.chiarello@unito.it (M.C.); claudio.baggiani@unito.it (C.B.)
[2] Institute of Agricultural Chemistry and Food Technology, Spanish Council for Scientific Research (IATA-CSIC), Paterna, 46980 Valencia, Spain; aabad@iata.csic.es (A.A.-F.); jvmercader@iata.csic.es (J.V.M.)
[3] Department of Organic Chemistry, University of Valencia, Burjassot, 46100 Valencia, Spain; antonio.abad@uv.es
[4] Unit of Infectious Diseases, Department of Medical Sciences, University of Turin, 10126 Turin, Italy; antonio.davolio@unito.it (A.D.); amedeo.denicolo@unito.it (A.D.N.)
* Correspondence: laura.anfossi@unito.it

**Contents**	**Page**
Materials and Chemicals	S1
AuNP synthesis	S1
Salt-induced aggregation stress test	S1
Figures	S2, S3

Materials and Chemicals

Tenofovir (TFV), disoproxil fumarate (TDF) and alafenamide (TAF) analytical standards were purchased by Selleck Chemicals (Houston, TX, USA). Elvitegravir was provided by Advanced ChemBlocks Inc (Burlingame, CA, USA), Dolutegravir by Ark Pharm (Arlington Heights, IL, USA), Adenosine triphosphate (ATP) by BDH Biochemical (Basel, CH) and Caffeine (Fluka, Charlotte, USA). Anti-TFV antibody, BSA-TFV and OVA-TFV antigens were produced by hAþtenics Group (Valencia, Spain). Peroxidase labelled rabbit anti-mouse immunoglobulin polyclonal antibody (RAM-HRP) was obtained from Dako (Glostrup, Denmark) and 3,3′,5,5′-tetramethylbenzidine from (Fluka, Charlotte, USA). To develop the standalone lateral flow for saliva were used the following materials. Gold (III) chloride trihydrate (ACS reagent), sucrose, bovine serum albumin (BSA), anti-mouse polyclonal antibody from rabbit, and the rest of the reagents and solvents were acquired from Merck (Darmstadt, Germany) and utilized without purification. Tween20 was purchased from VWR International (Milan, Italy). Nitrocellulose membranes (NC) CNPH 150 SS40-L2-P25 and Glass Fiber Sample Pad (GFBR4) were from MDI membrane technologies (Ambala, India). Glass fiber conjugate pad were purchased by Merck Millipore (Billerica, MA, USA). Color measurements and processing was made by using ImageJ software and

statistical calculations were carried out with SigmaPlot 14.0 software (Systat, Palo Alto, CA, USA).

AuNP synthesis

All the glassware involved in AuNP synthesis was previously washed with aqua regia (HNO3+HCl 1+3) to avoid any solid inorganic residue, avoiding any operation causing eventual scratches on the surface. The synthesis approach was based on the Turkevich/Frens method [28]. AuNPs with different sizes 32nm were prepared by reducing 2.94×10^{-5} mol of chloroauric acid with 4.93×10^{-6} mol of trisodium citrate dihydrate in boiling milliQ water refluxing with a six-bulb condenser. The localised surface plasmon resonance (LSPR) maximum wavelength was 525.5 nm and was acquired by means of a Cary 60 UV-Visible spectrophotometer (Agilent, CA, USA).

Salt-induced aggregation stress test

To define the amount of anti-TFV mAb that stabilizes AuNP, the salt-induced aggregation stress test was applied [29]. Therefore, 250 µl of AuNP solution at optical density 1 were inserted in wells of a microtiter plate and incubated for 30 min with increasing amounts (0-2.5µg) of the mAb. Then, 25 µl of aqueous NaCl (10% v/v) were added and reacted for 10 min to promote aggregation of unstable AuNPs. The absorbance of the solutions was read at 540 and 620 nm by a microplate reader (Multiskan FC, Microplate Photometer). The stabilizing amount of mAb was 8µg for each mL of AuNP at optical density 1 (Figure S3).

Accelerated aging study

The cassettes packaged in plastic bag with desiccant bags were incubated at 37 °C in the dark for 7 days. Testing was performed on day 3 and 7 by applying the pool of blank saliva sample unfortified and fortified at 0.25 -0.5 -1 ng/ml of TFV. The devices were tested in triplicate for each TFV concentration. Visual score readings and digital analyses were carried out as described. Data obtained on day 3 and 7 were compared to the one provided by the devices at their fabrication (day 0) by the one-way analysis of variance (Sigmaplot 12.0, Systat Software, CA, USA), a P-value less than 0.05 was considered for statistical significance.

FIGURES

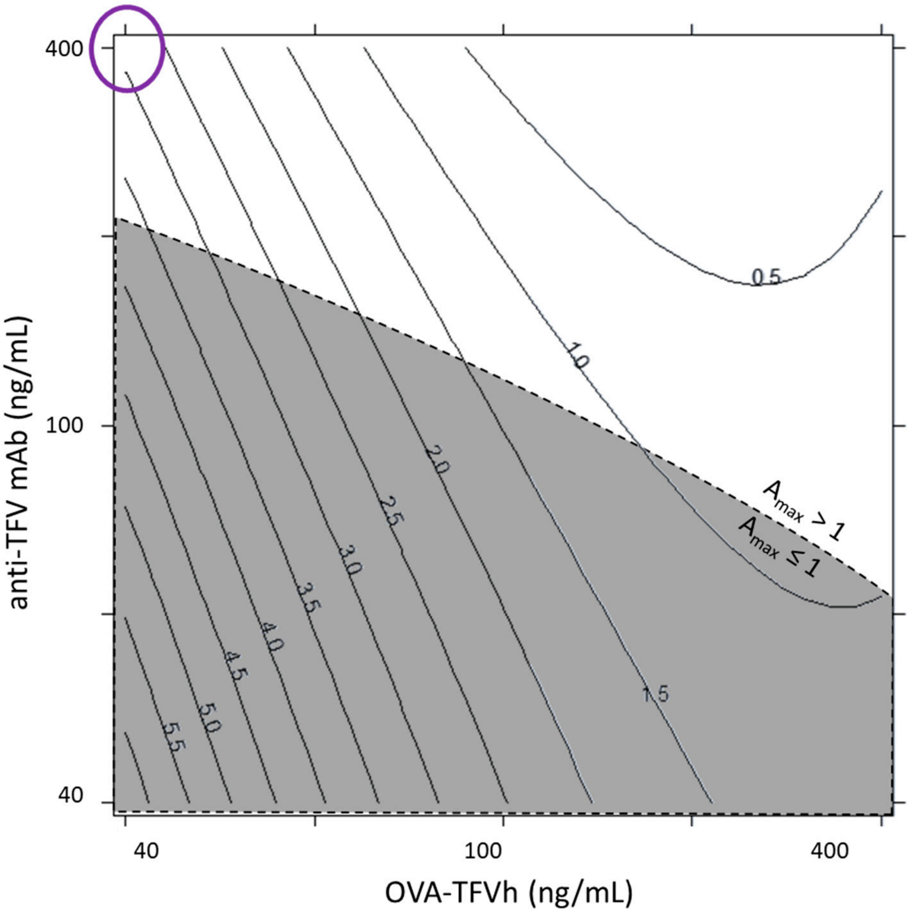

Figure S1. The contour plot obtained for the different amounts of anti-TFV mAb and coating antigen OVA–TFVh with a 1:5000 dilution of secondary labelled antibody RAM-HRP. The levels indicate the ratio between the maximum signal (Amax) and the half-maximal inhibitory concentration (IC50). Grey area includes unacceptable Amax≤1 results.

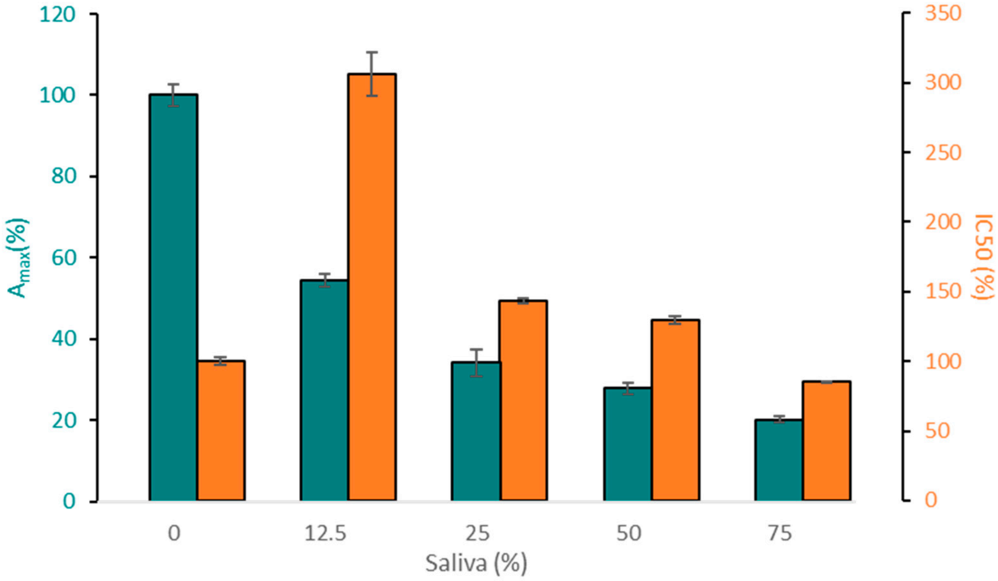

Figure S2. Matrix effect of different proportions of saliva on A_{max} and IC_{50} values of the cELISA based on anti-TFV mAb. Each value represents the mean of three determinations carried out on different days.

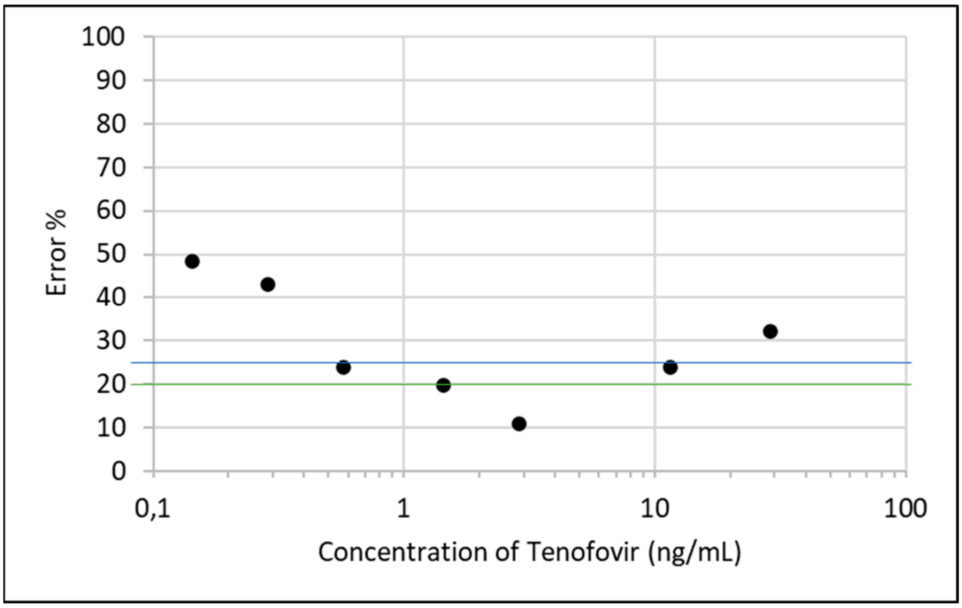

Figure S3. Plot of the error curve for the cELISA of salivary TFV**.** Percental error was calculated from the estimated TFV concentrations of calibrators as follows: (estimated TFV - theoretical value) / theoretical value *100. The mean error of 4 (intra-day) x 3 (inter-day) repeated measurements was plotted towards TFV concentration. The LOD, LLOQ and ULOQ were defined as the TFV concentrations that could be measured with an acceptable error (below 25% for the LOD, blue line, and below 20% for the LLOQ and ULOQ, green line).

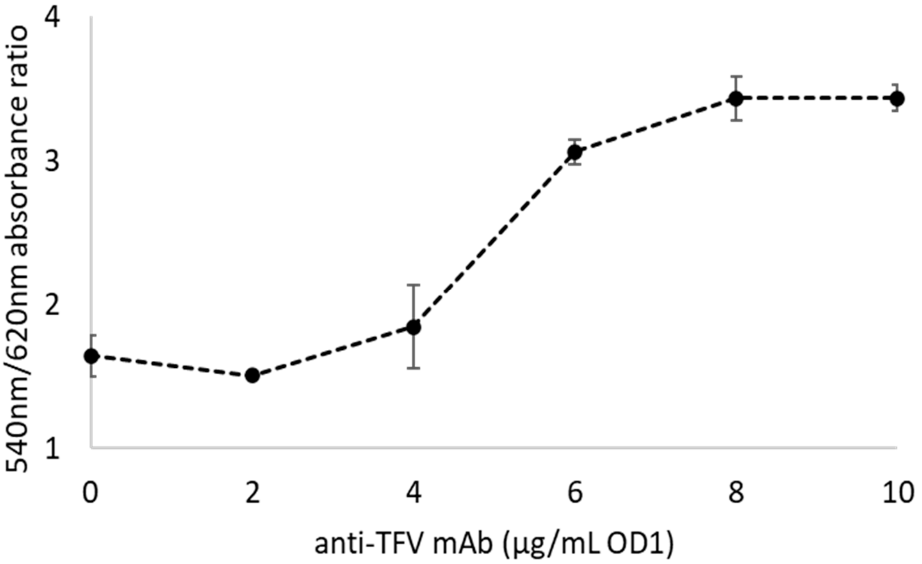

Figure S4. Salt-induced aggregation test on anti-TFV mAb on AuNPs of optical density of ca1.

Table S1. Stability of the LFIA device over time assessed by the thermal stress (7 days at 37°C). The LFIA for TFV were fabricated and stored in a plastic bag containing silica and in the dark. The colour of the Test line was measured after applying a blank saliva sample (in triplicate) on the day of LFIA fabrication and after 3 and 7 days. The vLOD was determined as the concentration of TFV that completely inhibited the colouring of the Test line. .

days@37°C	Test line colour mean±SD (a.u.)	vLOD[1] (ng/mL)
**0**	89±6[2]	0.5
**3**	77±8[2]	0.5
**7**	81±6[2]	0.25-0.5

References

28. Kimling, J.; Maier, M.; Okenve, B.; Kotaidis, V.; Ballot, H.; Plech, A. Turkevich Method for Gold Nanoparticle Synthesis Revisited. *J. Phys. Chem. B* **2006**, *110*, 15700–15707. https://doi.org/10.1021/jp061667w.

29. Cavalera, S.; Pezzoni, G.; Grazioli, S.; Brocchi, E.; Baselli, S.; Lelli, D.; Colitti, B.; Serra, T.; Di Nardo, F.; Chiarello, M.; et al. Investigation of the "Antigen Hook Effect" in Lateral Flow Sandwich Immunoassay: The Case of Lumpy Skin Disease Virus Detection. *Biosensors* **2022**, *12*, 739. https://doi.org/10.3390/bios12090739.
